# S-Pen Technology and Online Signatures: Cross-Device Variability and Its Implications for Mobile Biometric Authentication

**DOI:** 10.3390/s26051451

**Published:** 2026-02-26

**Authors:** Gerardo Reyes-García, Abel Garcia-Barrientos, Ernesto Zambrano-Serrano, Ignacio Algredo-Badillo

**Affiliations:** 1 Faculty of Science, Universidad Autónoma de San Luis Potosí, San Luis Potosí 78217, Mexico; 2 Facultad de Ingeniería Mecánica y Eléctrica, Universidad Autónoma de Nuevo León, San Nicolás de los Garza 66455, Mexico; ernesto.zambranos@uanl.edu.mx; 3 Computer Science Department, SECIHTI-INAOE, Tonantzintla 72840, Mexico; algredobadillo@inaoep.mx

**Keywords:** online signature verification, behavioral biometrics, cross-device variability, Dynamic Time Warping, mobile devices, stylus-based input

## Abstract

This paper presents a pilot study on cross-device variability in online signature dynamics captured on consumer Samsung devices using S-Pen technology. Signature data were acquired on two devices, a Galaxy Ultra smartphone and a Galaxy Tab S6 Lite tablet, through a unified web-based interface designed to ensure consistent capture across platforms. The acquisition process recorded timestamped x–y trajectories, stroke events, and pressure information when available, preserving temporal structure for dynamic analysis. Genuine signatures were systematically divided into reference and test sets, and comparisons were performed under intra-device conditions (enrollment and verification on the same device) and cross-device conditions (enrollment and verification on different devices). Similarity was evaluated using Dynamic Time Warping (DTW) on multivariate time series, with analysis focused on how differences in form factor and writing area influence signature behavior. This problem is directly relevant to mobile biometric authentication workflows, where users frequently enroll on one device and later verify on another; under this mismatch scenario, reduced separability between genuine and impostor scores can affect decision reliability. Consistent with this interpretation, the results show lower dissimilarity in intra-device comparisons and higher distances with ROC degradation under cross-device mismatch. These findings provide exploratory evidence that device heterogeneity is a practical factor in mobile signature verification and support the need for cross-device-aware design in authentication systems used for digital transactions and document authorization in real-world mobile environments.

## 1. Introduction

The online signature verification is one of the most extensively studied behavioral biometric modalities, owing to its long-standing social acceptance, ease of acquisition, and relatively low hardware requirements [1,2,3,4,5,6,7,8,9]. Signatures have historically served as a legally binding means of identity verification in financial, administrative, and legal contexts, which has facilitated their transition into the digital domain [10,11,12,13,14,15,16,17,18,19,20,21]. Unlike physiological biometrics such as fingerprints, iris patterns, or facial features, signatures are behavioral in nature and therefore reflect not only physical characteristics but also neuromotor processes and learned motor patterns [22,23,24,25,26,27,28,29,30]. This dual nature makes online signatures both appealing and challenging as a biometric trait [7,8].

In online (also referred to as dynamic) signature verification, the signing process is recorded as a time-dependent sequence of events rather than as a static image [7,8,12]. Typical captured information includes spatial coordinates of the pen tip over time, velocity and acceleration profiles, stroke segmentation through pen-up and pen-down events, and, when available, additional channels such as pressure, tilt, and azimuth [8,12]. These dynamic characteristics encode how a signature is produced rather than simply what it looks like [8,12,24]. As a result, online signatures often provide greater discriminatory power than offline signatures, which are limited to static visual appearance [8,12]. Numerous studies have demonstrated that skilled forgeries may visually resemble genuine signatures while failing to reproduce the temporal structure and motor dynamics of the signing process [7,8].

Despite these advantages, online signature verification remains a complex problem due to the intrinsic variability of human motor behavior [24,25,26]. Even under controlled conditions, genuine signatures from the same individual may exhibit substantial variation across signing sessions [24,25]. Factors such as fatigue, emotional state, stress, signing speed, posture, and environmental context can all influence the resulting signature dynamics [24,25]. This intra-user variability must be carefully modeled in any practical verification system to avoid excessive false rejections of genuine users [8,9]. At the same time, systems must remain sensitive enough to detect skilled forgeries, which may approximate spatial shape while differing subtly in timing or pressure patterns [7,8].

Beyond human-related variability, acquisition conditions play a crucial role in shaping the recorded signature signal [8,22]. The characteristics of the sensing device—including sampling frequency, spatial resolution, pressure sensitivity, and latency—can significantly affect the captured trajectories [22]. The physical properties of the writing surface, such as friction and texture, influence pen movement and stroke smoothness [25]. Additionally, the size and aspect ratio of the available writing area can alter the spatial scaling, stroke length, and layout of the signature [22,25]. Consequently, the same user signing on different devices, or even on the same device under different configurations, may produce signatures with markedly different dynamic properties [22].

Traditional online signature datasets and benchmark studies have often relied on controlled acquisition environments using dedicated digitizing tablets [1,2,3]. These tablets typically provide consistent sampling rates, high-resolution sensing, and a fixed writing area, thereby minimizing acquisition-related variability [1,2,3]. While such setups are well-suited for algorithm development and comparative evaluation, they do not fully reflect the conditions encountered in modern deployment scenarios [22].

In recent years, the landscape of online signature acquisition has shifted toward consumer-grade mobile devices, particularly smartphones and tablets equipped with active stylus input [22]. These devices are now widely used for electronic document signing, consent forms, contract approval, and mobile authentication workflows [22]. The increasing prevalence of stylus-enabled mobile devices introduces new challenges for online signature verification [22]. Consumer devices vary widely in form factor, screen size, ergonomics, and interaction modality [22]. Smartphones typically offer a relatively small writing area and are often held in one hand while signing with the other, potentially leading to constrained or compressed signature movements [22]. Tablets, by contrast, provide larger screens and may be placed on a table or held at different angles, allowing for broader arm movements and altered wrist dynamics [25]. Even when devices share a common stylus technology ecosystem, such as Samsung’s S-Pen, differences in hardware implementation and usage context can introduce systematic variations in the captured signature data [22].

In real-world authentication scenarios, it is increasingly common for users to enroll their signature on one device and later be required to verify it on another [22]. For example, a user may initially provide a reference signature on a tablet during an onboarding or registration process and subsequently authenticate transactions on a smartphone while on the move [22]. This cross-device usage pattern is fundamentally different from the assumptions underlying many traditional verification systems, which implicitly assume consistent acquisition conditions between enrollment and verification [22]. As a result, understanding cross-device variability has become a critical issue for the practical deployment of online signature verification systems [22].

Cross-device variability arises from multiple interacting factors [22]. Differences in screen size and writing area may cause signatures to be spatially scaled or compressed [22]. Changes in device orientation, grip, and posture can affect stroke direction, velocity, and smoothness [25]. Sampling rates and sensor noise characteristics may differ between devices, altering the temporal resolution of the captured signal [22]. Moreover, users may unconsciously adapt their signing behavior to the device, resulting in systematic changes in signing style [25]. Together, these factors contribute to mismatches between signatures acquired on different platforms, potentially increasing intra-user distances and degrading verification performance [22]. Despite its practical importance, cross-device effects in online signature verification remain relatively underexplored in consumer mobile devices [22]. Many existing studies focus on improving accuracy within a single device or dataset, without explicitly examining generalization across heterogeneous platforms [22]. Therefore, as mobile authentication expands, empirical studies are needed to characterize the impact of device heterogeneity on signature dynamics and similarity measures [22]. Table 1 summarizes representative prior work in online signature verification and highlights whether cross-device effects are explicitly considered.

The present work addresses this gap by investigating cross-device variability in online signature dynamics acquired using consumer-grade Samsung devices with S-Pen input: a Galaxy Ultra smartphone and a Galaxy Tab S6 Lite tablet [22]. These devices were selected to represent two commonly used mobile form factors with substantially different screen sizes and interaction areas, while maintaining a consistent stylus technology and operating ecosystem [22]. By focusing on devices that are widely available to consumers, this study aims to provide insights that are directly relevant to real-world mobile authentication scenarios [22]. To facilitate flexible and device-independent data collection, a lightweight web-based acquisition interface was developed. Unlike native applications tailored to a specific platform, a web-based interface enables consistent deployment across multiple devices with minimal configuration overhead. Each signature is captured as a structured multivariate time series, preserving temporal ordering and spatial information. This representation allows for direct analysis of dynamic properties and supports the application of sequence-based similarity measures. The use of a standardized data format also simplifies cross-device comparison and future dataset extension. Similarity between signatures is evaluated using DTW-based similarity analysis; details on DTW computation and the length-normalized DTW score are provided in Section 3.3 [20,29]. In summary, this work investigates the impact of device heterogeneity on online signature dynamics by analyzing signatures captured on a smartphone and a tablet using a consistent stylus ecosystem and a web-based acquisition interface [22]. Through DTW-based similarity analysis under intra-device and cross-device conditions, the study explores how differences in form factor and writing area influence dynamic signature behavior [22,25]. By focusing on consumer mobile devices and real-world usage considerations, the study aims to contribute to a more realistic understanding of the challenges facing online signature verification systems and to provide guidance for future research in this increasingly important application domain [22].

From a biometric security perspective, this cross-device setting defines an operational risk surface: a user enrolled on one device and verified on another may produce higher genuine-score variability, which can increase overlap with impostor-score distributions and complicate threshold selection. In practical terms, this mismatch can raise false-rejection risk for legitimate users and, depending on operating point, may also affect false-acceptance behavior. Therefore, in this pilot study, cross-device variability is treated as a security-relevant source of uncertainty in mobile signature authentication rather than only as a usability or signal-processing issue.

## 2. Materials and Methods

This study adopts an exploratory experimental methodology to analyze cross-device variability in online signature dynamics acquired from consumer-grade mobile devices [22]. The methodology is designed to reflect realistic mobile signing conditions while maintaining sufficient structure to enable meaningful comparison between devices [22]. It encompasses participant recruitment, data acquisition setup, signature capture protocol, data representation, and similarity analysis.

Online signatures were collected from 15 adult participants recruited on a voluntary basis. Each participant provided 10 genuine signatures per device, using both the Galaxy Ultra smartphone and the Galaxy Tab S6 Lite tablet, resulting in a total of 300 genuine signature samples across the study. Before acquisition, participants received the same brief operational instructions regarding how to complete the signing task, and the capture process was executed through a unified web-based interface to maintain procedural consistency across devices. Data collection was conducted under the same protocol to reduce avoidable acquisition bias and preserve comparability between intra-device and cross-device analyses. The participant pool was intentionally heterogeneous in terms of handwriting style, stroke dynamics, signing habits, writing speed, and degree of familiarity with stylus-based interaction. This heterogeneity was retained to reflect realistic usage conditions and to avoid an artificially constrained cohort that could mask natural behavioral variability [24,25,26]. In practical terms, some participants reported frequent use of tablets or pen-enabled screens in academic or professional contexts, while others had limited prior exposure to digital pen input and required short adaptation at the start of the task. Preserving this range of user profiles was considered important for evaluating cross-device effects under conditions closer to everyday mobile authentication scenarios rather than under highly controlled laboratory behavior. This design choice supports the exploratory objective of assessing whether a device change alone can alter signature dynamics in a way that impacts downstream verification behavior. Consequently, the dataset was structured to allow direct comparison of the same-user signatures across same-device and different-device conditions while maintaining a consistent collection framework. To preserve participant privacy and comply with ethical considerations, each subject was assigned an anonymous identifier (P01, P02, etc.) consistently throughout data storage and analysis. No personally identifiable information, such as names, demographic attributes, or biometric identifiers beyond the signatures themselves, was collected or stored. All participants were informed of the purpose of the study and provided consent prior to data acquisition.

**Figure 1:**
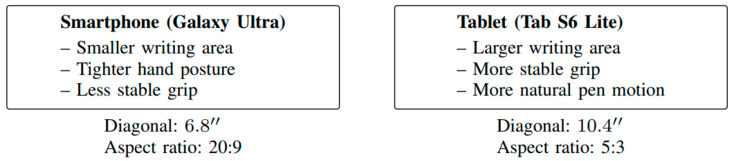
Comparison of the smartphone and tablet used for data acquisition, illustrating differences in writing area, ergonomics, and screen dimensions.

Signature data were acquired using two consumer mobile devices equipped with Samsung S-Pen technology: a Samsung Galaxy Ultra smartphone and a Samsung Galaxy Tab S6 Lite tablet [22]. These devices were selected to represent two commonly used mobile form factors with substantially different screen sizes and interaction characteristics, while maintaining a consistent stylus ecosystem [22]. Both devices support active pen input with pressure sensitivity and provide system-level access to stylus event data through web-based interfaces. The smartphone and tablet differ in several aspects that are relevant to signature acquisition, including writing surface area, screen resolution, physical dimensions, and typical usage posture [22]. The Galaxy Ultra smartphone provides a relatively small signing area and is often held in one hand while signing with the other, potentially constraining movement and encouraging wrist-dominant strokes [22]. In contrast, the Tab S6 Lite tablet offers a larger writing surface and is more likely to be placed on a table or held with both hands, allowing broader arm movements and different stroke dynamics [25]. These differences are representative of common real-world conditions in which users may alternate between devices for signing tasks [22]. A visual comparison of the devices and their relative writing areas is provided in Figure 1.

**Figure 2:**
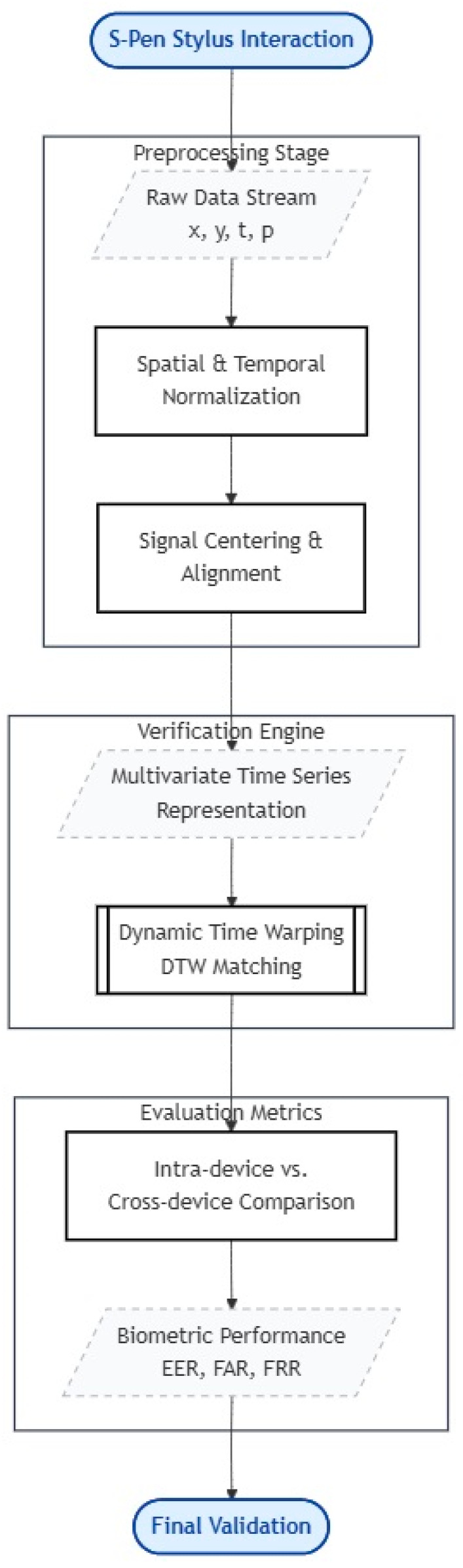
Block diagram of the proposed methodology, including signature acquisition, data recording, time-series construction, and DTW-based similarity analysis.

To ensure platform independence and consistency across devices, a lightweight web-based signature acquisition interface was developed. This choice enables the same acquisition workflow, see Figure 2, to be deployed across multiple consumer devices with minimal configuration overhead, avoiding differences introduced by device-specific native applications. The interface records each signature as a structured multivariate time series while preserving temporal ordering and spatial information, supporting consistent downstream preprocessing and DTW-based comparison.

The interface runs in a standard mobile web browser and captures stylus input events in real time without requiring device-specific native applications [22]. This design choice reduces implementation complexity and facilitates reproducibility while allowing the same acquisition logic to be deployed on both devices [22]. The interface presents participants with a blank signing area occupying a fixed proportion of the available screen space. Participants were instructed to sign naturally within this area using the S-Pen, without attempting to adjust their signature style to the device. During signing, the interface records a sequence of stylus events, including pen-down, pen-move, and pen-up actions. Each event is timestamped and associated with spatial coordinates relative to the signing area. When supported by the device and browser, additional attributes such as pressure are also recorded. Each captured signature is stored as a structured multivariate time series, preserving the temporal ordering of events. This representation allows direct analysis of dynamic properties and supports sequence-based similarity measures such as Dynamic Time Warping [20,29]. All signature data are stored locally in a structured format and later exported for offline analysis. Participants were asked to provide multiple genuine signature samples on each device. The signing sessions were conducted in a casual indoor environment to approximate everyday usage rather than a strictly controlled laboratory setting [22]. Participants were allowed to sit comfortably and hold the device in a manner they found natural, reflecting realistic mobile signing behavior [22]. No constraints were imposed on signing speed or pressure, and participants were encouraged to sign as they normally would when authorizing documents. To minimize short-term memory effects while keeping the protocol practical, signatures on the two devices were collected in separate sessions, with a brief pause between device changes. The order of device usage was kept consistent across participants to reduce procedural variability, though the study does not attempt to eliminate all ordering effects. Each signature was treated as an independent sample, and no feedback was provided to participants regarding signature quality or consistency. The resulting dataset contains multiple signatures per participant per device, enabling both intra-device comparisons (signatures from the same participant on the same device) and cross-device comparisons (signatures from the same participant across different devices) [22]. In operational terms, this setting represents a common mobile-authentication mismatch condition in which enrollment and verification may occur on different platforms. Due to the pilot-scale nature of the study, the number of participants and signatures per participant is limited; however, the dataset is sufficient to support exploratory analysis of device-related effects and their potential impact on score separability under cross-device use [22].

**Figure 3:**
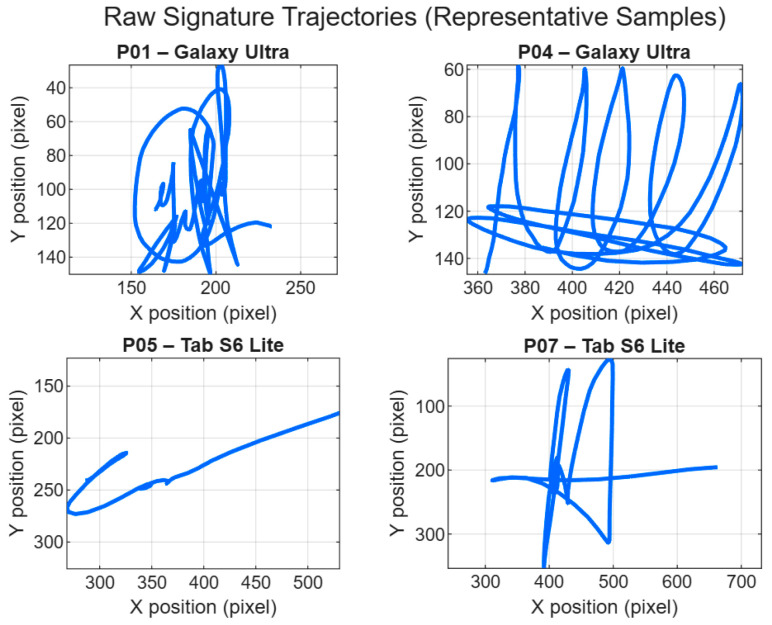
Raw signature trajectories of four representative participants acquired on smartphone (Galaxy Ultra) and tablet (Tab S6 Lite) devices using S-Pen input; all subplots are displayed with a common axis scale to preserve visible differences in effective writing area between devices.

Figure 3 illustrates representative raw signature trajectories acquired from multiple participants on the smartphone (Galaxy Ultra) and tablet (Tab S6 Lite) devices, highlighting both inter-user and inter-device variability in raw form for visualization (prior to the per-signature z-score normalization of x and y applied only for DTW comparison, Section 3.1) and without any resampling. The plotted traces preserve the original temporal–spatial behavior captured during acquisition, including natural differences in stroke curvature, trajectory scale, and local shape complexity across users. In addition, noticeable device-dependent effects can be observed, such as changes in overall spatial extent and stroke spacing that are consistent with differences in writing area and interaction mechanics between the two platforms. These qualitative observations provide an intuitive motivation for the subsequent DTW-based comparisons, since DTW is designed to accommodate temporal misalignment while still reflecting geometric discrepancies when device mismatch is present [20,22,23].

Raw signature samples are stored as multivariate time series extracted from CSV files generated by the acquisition interface. Each signature is represented as an ordered sequence of points, where each point contains the x-position (pixels), y-position (pixels), and the stylus pressure value when available. To ensure consistency across samples while preserving device-induced variability, only minimal preprocessing is applied: (i) verification of temporal ordering and (ii) removal of duplicate consecutive samples. No filtering, smoothing, or resampling is performed. However, prior to DTW comparison, x and y are standardized per signature using z-score normalization (zero mean, unit variance), as described in Section 3.1, while pressure is kept in its native scale when available. This choice keeps the analysis aligned with raw acquisition behavior while preventing trivial coordinate offsets/scale from dominating the DTW cost [20,23].

Signature similarity is measured using multivariate Dynamic Time Warping (DTW), which aligns two sequences under non-linear temporal warping to account for differences in signing speed and local timing variations [20,29]. DTW distances are computed across four evaluation conditions: intra-device (phone-to-phone and tablet-to-tablet) and cross-device (phone-to-tablet and tablet-to-phone) [22]. The resulting DTW distance serves as a measure of dissimilarity between signatures; lower distances indicate greater similarity. Rather than defining a verification threshold or reporting classification accuracy at this stage, the analysis focuses on comparing DTW distance distributions across these conditions, consistent with the exploratory goal of assessing device-induced variability. Details on DTW computation and the length-normalized DTW score are provided in Section 3.3 [20,29].

Subsequent sections detail dataset preparation and preprocessing (Section 3.1) and DTW-based analysis (Section 3.3 and Section 4) [22]. It begins with signature acquisition on two mobile devices using a unified web-based interface, followed by structured time-series representation and minimal preprocessing. DTW-based similarity analysis is then performed to compare intra-device and cross-device signature pairs. The resulting distance measures are analyzed to identify trends and differences attributable to device form factor and writing area [22]. By combining realistic mobile acquisition conditions with a transparent and reproducible analysis pipeline, this methodology provides a foundation for understanding cross-device effects in online signature verification [22]. While limited in scale, the approach is designed to highlight key challenges and inform the design of future, larger-scale studies in heterogeneous mobile environments [22].

## 3. Experimental Setup

### 3.1. Dataset Preparation

Figure 3 provides representative raw signature trajectories for visualization; dataset preparation and the per-signature z-score normalization of x and y (applied only at DTW comparison time) are described in this section [20,22,23]. For each participant, 10 signatures were collected per device (smartphone and tablet), yielding two device-dependent subsets per subject. All samples were visually inspected to ensure completeness and consistent stroke formation before inclusion in the experimental dataset, and obvious acquisition artifacts (e.g., truncated traces or unintended interruptions) were excluded when identified. This manual screening step helps ensure that the subsequent DTW comparisons reflect device-related variability rather than data recording errors.

### 3.2. Reference and Test Splits

Following common evaluation practices in online signature verification—where a subset of signatures is used for enrollment (reference templates) and the remaining samples are used for testing—each participant’s dataset was divided into [1,2,8,23]:

Reference set: An enrollment subset (used as reference templates).

Test set: A disjoint subset (used for similarity evaluation).

For each participant and for each device, 5 signatures were assigned to the reference set, and the remaining 5 signatures were assigned to the test set.

Two evaluation conditions were examined:

1. Intra-device evaluation. Training and testing were conducted on the same device (Smartphone to Smartphone and Tablet to Tablet). This setting reflects the conventional writer-dependent scenario, where device-consistent acquisition typically yields lower dissimilarity scores.
2. Cross-device evaluation. Training and testing were conducted across devices (Smartphone to Tablet and Tablet to Smartphone). Prior work on mobile/device-dependent acquisition reports performance degradation and feature/trajectory distortions due to differences in screen size, writing ergonomics, and sensor characteristics; therefore, higher DTW distances were anticipated under these conditions [8,22,23].

### 3.3. Feature Extraction and DTW-Based Similarity

For each signature, a multivariate time series was constructed using the x-position (pixels), y-position (pixels), and pressure level when available. This raw trajectory representation is consistent with standard formulations in dynamic signature verification, where temporal handwriting signals are directly exploited for sequence comparison [7,8,11,12,15,23]. In this pilot study, similarity analysis is performed using a deterministic DTW-based framework, and no learning-based model is trained; therefore, the methodology should be interpreted as non-learning analytical rather than AI-driven classification.

Dynamic Time Warping (DTW) was employed to measure similarity between reference and test signatures across all evaluation conditions. DTW computes the minimum accumulated alignment cost between two time series under non-linear temporal warping, enabling alignment of signatures with different lengths and signing speeds [20,29]. In addition to raw DTW distances, a length-normalized DTW score was computed to reduce the influence of sequence length on the similarity measure. The normalized score was obtained by dividing the accumulated *DTW* cost by the length of the optimal warping path:

$$DTWnorm=\frac{DTW}{L}$$

where *DTW* denotes the accumulated alignment cost and *L* is the length of the corresponding optimal warping path. This normalization is applied only at the comparison stage and does not modify the raw input trajectories, which remain unfiltered and unresampled, except for the per-signature z-score normalization of x and y described in Section 3.1 [20,23].

## 4. Results

This section presents the experimental results obtained from the proposed cross-device online signature analysis framework. Accordingly, the reported outcomes are framed as analytical evidence from distance-based comparison, not as results from a trained machine-learning model. Signature data were collected from multiple participants using two consumer devices equipped with S-Pen input (a smartphone and a tablet). The results combine qualitative visualization and quantitative evaluation in order to capture both interpretable device effects and measurable verification performance under heterogeneous acquisition. Specifically, results are reported at three complementary levels: (i) group-level distance trends summarizing intra-device versus cross-device DTW behavior, (ii) ROC-based verification performance under different enrollment/verification device pairings, and (iii) participant-level distance distributions that illustrate individual variability and device dependency. DTW-based distances are used as the primary similarity measure, and normalized DTW scores are employed for verification analysis to reduce the influence of sequence length at comparison time [20,29]. In addition to aggregate curves and distributions, representative examples are included to provide intuition about how trajectory shape, scale, and stroke spacing change across devices prior to DTW-based comparison. All similarity measurements and verification metrics are computed using genuine comparisons (reference vs. test signatures from the same user) and impostor comparisons (signatures from different users), following standard biometric verification practice in online signature evaluation [8,9,23].

### 4.1. Group-Level Analysis

**Figure 4:**
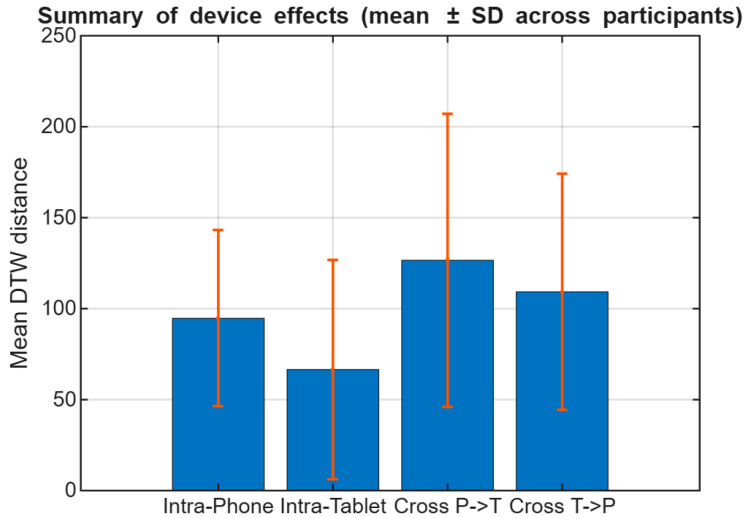
Mean Dynamic Time Warping (DTW) distances ± standard deviation for the illustrated subset of participants (P01, P04, and P05) under intra-device and cross-device evaluation conditions.

**Figure 5:**
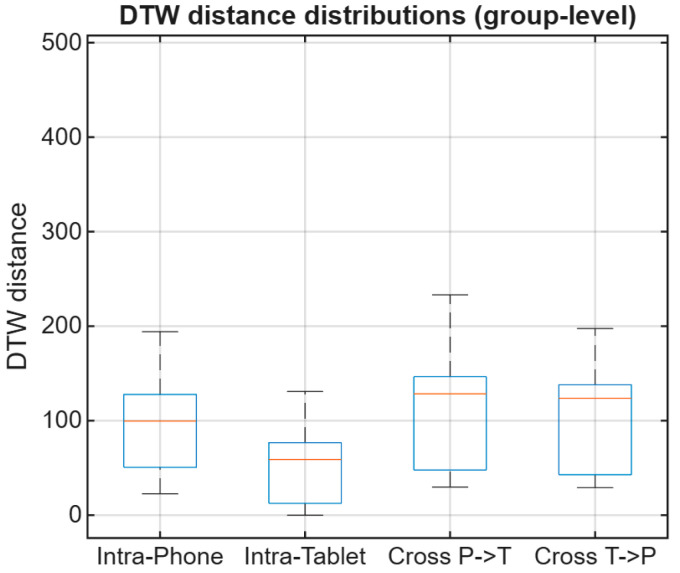
Group-level DTW distance distributions (boxplots) for participants P01, P04, and P05 under intra-device and cross-device evaluation conditions.

Across participants, intra-device comparisons yield lower DTW distances than cross-device comparisons, indicating higher similarity when signatures are captured and compared on the same device. This group-level analysis provides a descriptive characterization of how DTW alignment costs are affected by device changes, highlighting systematic shifts in distance magnitude between intra-device and cross-device conditions. These aggregated trends offer an intuitive overview of device-induced variability; as an illustration, Figure 4 summarizes mean DTW distances ± standard deviation for a subset of participants. Participant P07 is included in Figure 3 as a qualitative trajectory illustration, whereas the compact subset displayed in Figure 4 and Figure 5 (P01, P04, and P05) was selected only for visualization clarity.

It should be noted that this analysis is descriptive in nature, while formal verification performance is evaluated separately through ROC-based biometric analysis in the subsequent subsection.

Unless otherwise stated, cohort-level summary trends and verification metrics are computed using data from all participants in the study, while the reduced participant subset in Figure 4 and Figure 5 is used only to maintain visual readability.

In addition to mean trends, the distribution of DTW distances highlights increased dispersion under cross-device conditions, as shown in Figure 5. Similar device-related performance/feature robustness effects have been reported in mobile online signature verification studies and surveys [6,8,22,23].

Although Figure 4 provides a compact mean ± standard deviation summary, it may hide differences in spread and overlap between conditions. Therefore, we also examine the full DTW distance distributions in Figure 5 to better characterize dispersion and tail behavior under device mismatch.

### 4.2. ROC-Based Verification Analysis

To complement the distance-based analysis, biometric verification performance was evaluated using Receiver Operating Characteristic (ROC) curves. Genuine scores were computed by matching reference and test signatures from the same participant, while impostor scores were obtained by comparing test signatures against reference sets from other participants, following standard biometric verification protocols [8,9,23]. This evaluation characterizes performance across decision thresholds and provides a clearer view than average distances when score distributions overlap [8,9].

**Figure 6:**
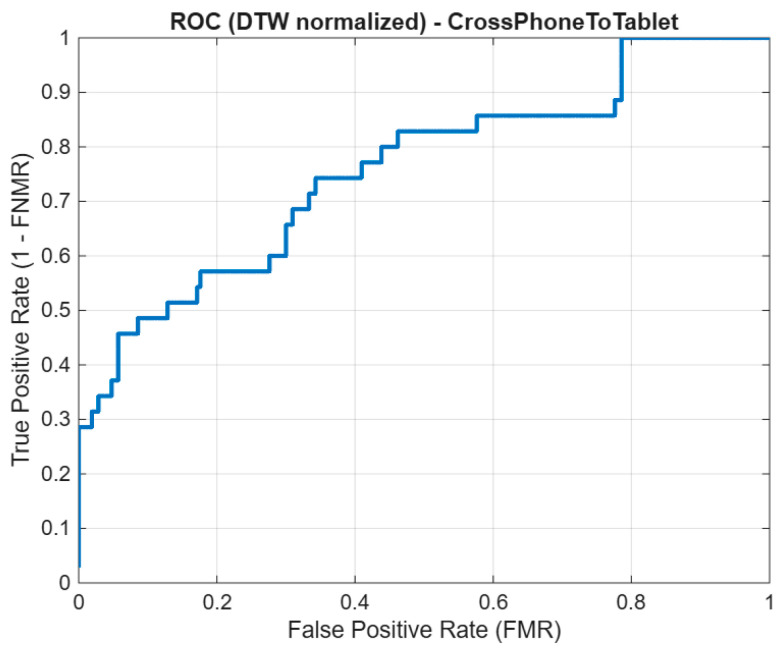
ROC curve for cross-device signature verification (Phone to Tablet) using the length-normalized DTW score.

ROC curves were computed for intra-device and cross-device scenarios using the length-normalized DTW score defined in Section 3.3. DTW-based matching is widely used for aligning signatures with different lengths and signing rates [20,29]. While score normalization reduces dependence on sequence length, it does not remove geometric distortions introduced by device mismatch, which can dominate the alignment cost when writing area and interaction mechanics differ [8,22,23]. Figure 6 shows the ROC curve for the Phone to Tablet condition; compared to intra-device scenarios, performance degrades (curve closer to the diagonal), indicating increased overlap between genuine and impostor distributions under device mismatch, consistent with prior reports on mobile/cross-device settings [6,8,22,23].

To complement the ROC interpretation with explicit operating metrics, threshold-independent and operating-point indicators were computed for both evaluation conditions. Under intra-device verification, the system reached AUC = 0.88, EER = 17.6%, and FNMR = 31.4% at FMR = 1%. Under cross-device verification, the corresponding values were AUC = 0.71, EER = 31.8%, and FNMR = 58.7% at FMR = 1%. The same degradation trend was observed at FMR = 5%, where FNMR increased from 18.9% (intra-device) to 39.6% (cross-device). These quantitative metrics are consistent with the ROC behavior and reinforce that device mismatch reduces separability and increases rejection risk for genuine users in practical mobile verification settings.

Given the pilot-scale nature of this study, the ROC analysis is intentionally presented as descriptive evidence of performance tendency rather than as a formal population-level statistical claim. Although the observed intra-device versus cross-device separation pattern is consistent across the analyzed comparisons, the cohort size and session depth remain limited for robust inferential testing at this stage. For this reason, hypothesis-testing procedures (e.g., non-parametric significance tests over score distributions) were not applied in the present version, and confidence intervals for ROC-derived operating behavior were not estimated. This methodological boundary should be considered when interpreting the strength of the reported degradation trend under device mismatch. In practical terms, the results support a directional conclusion—reduced separability in cross-device conditions—while the exact effect magnitude should be interpreted with caution in broader deployment contexts. Therefore, the current ROC findings are positioned as pilot-level analytical evidence that motivates larger follow-up studies with expanded samples, more sessions per user, and explicit inferential validation. This interpretation is consistent with the exploratory scope of the manuscript and avoids overgeneralization beyond the evaluated acquisition setting.

### 4.3. Participant-Level Analysis

**Figure 7:**
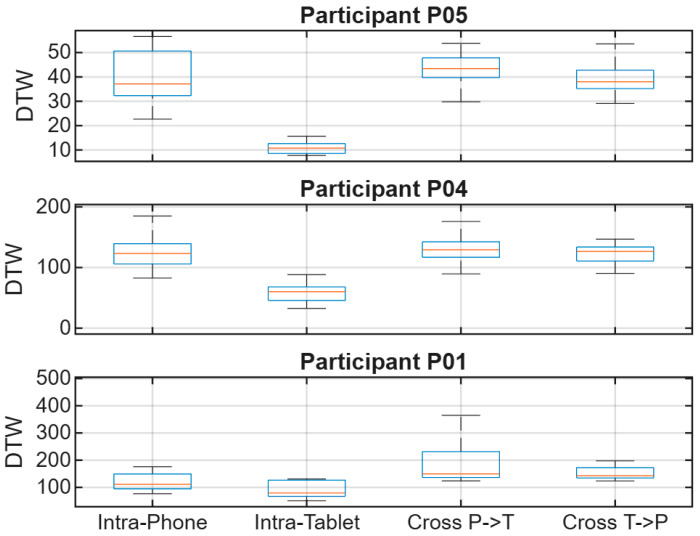
DTW distance distributions for participants P01, P04, and P05 across intra-device and cross-device evaluation conditions.

Figure 7 presents participant-level DTW distance distributions under intra-device and cross-device conditions. Instead of a single mean value, these distributions show spread and overlap across trials and reveal whether device mismatch produces a consistent upward shift or mainly increases dispersion. This view highlights individual differences in device dependency and supports the participant-specific observations discussed below.

#### 4.3.1. Participant P01

**Figure 8:**
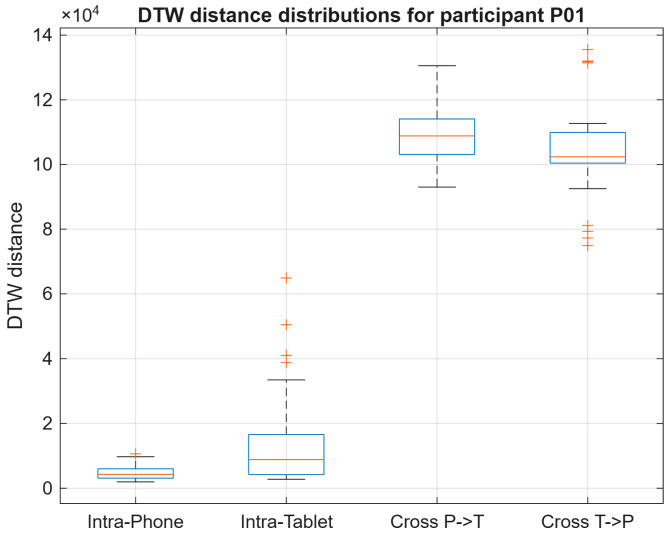
DTW distance distributions for participant P01 across all evaluation conditions.

As shown in Figure 8, participant P01 exhibited strong device dependency. Intra-Phone distances were consistently low, indicating high signing stability on the smartphone, while Intra-Tablet distances were moderately higher. Cross-device comparisons showed a sharp increase in DTW distances, representing the largest device shift observed among the illustrated participants.

#### 4.3.2. Participant P04

**Figure 9:**
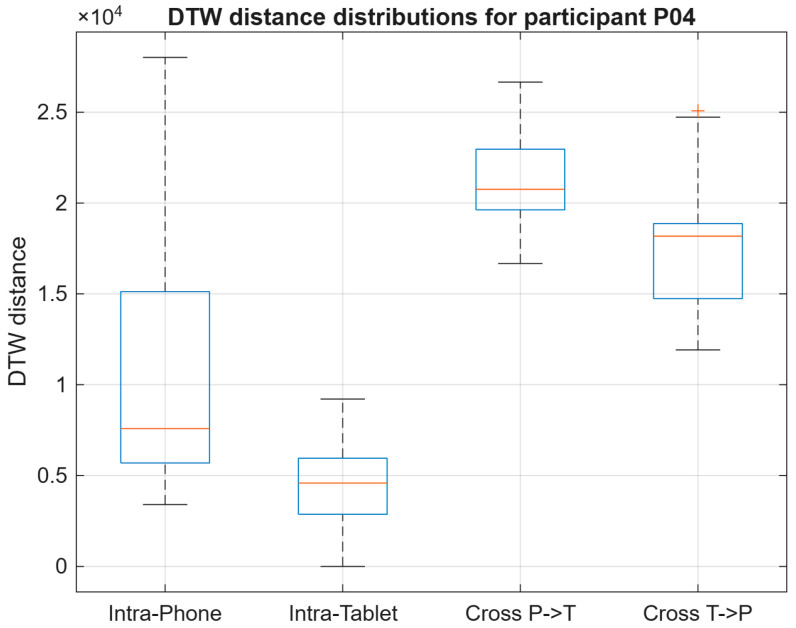
DTW distance distributions for participant P04 across all evaluation conditions.

As shown in Figure 9, participant P04 exhibited a more balanced profile across devices. Both Intra-Phone and Intra-Tablet DTW distances remained at moderate levels, suggesting relatively consistent signing behavior within each device. Cross-device distances increased as expected under device mismatch, but the separation from intra-device comparisons was less pronounced than for P01, indicating reduced device dependency for this participant. Overall, P04 illustrates an intermediate case where device effects are present but do not dominate the distance patterns, supporting the notion that cross-device variability can be strongly participant-dependent.

#### 4.3.3. Participant P05

**Figure 10:**
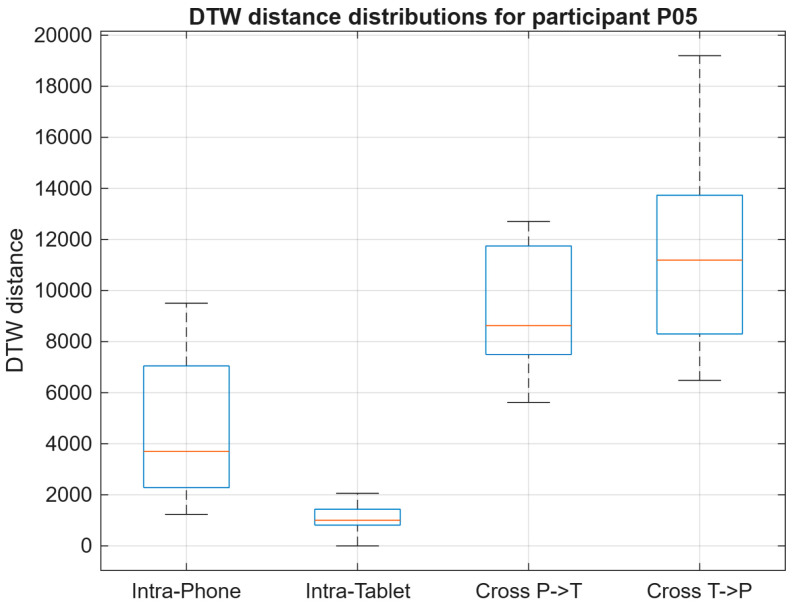
DTW distance distributions for participant P05 across all evaluation conditions.

As illustrated in Figure 10, participant P05 exhibited the lowest DTW distances under the Intra-Tablet condition, indicating higher signing stability on the larger writing surface. In contrast, Intra-Phone distances were more variable, while cross-device comparisons showed elevated DTW values, following the expected trend. This may indicate that the tablet allows smoother, less constrained strokes, whereas the smaller smartphone surface can amplify small variations in spacing and speed, increasing alignment cost. This qualitative pattern aligns with observations in mobile signature verification literature where acquisition conditions and device form factor can affect trajectory consistency and verification robustness [6,8,22,23].

### 4.4. Summary of Device Effects

Across the evaluated participants, the results indicate strong device dependency: signatures acquired on smartphones and tablets should not be assumed interchangeable without accounting for device mismatch. Cross-device DTW distances were consistently higher than intra-device distances, reflecting systematic differences likely induced by changes in writing area, interaction mechanics, and sensing characteristics. These findings are consistent with prior reports on feature robustness issues and performance degradation under mobile and cross-device acquisition conditions [6,8,22,23].

## 5. Discussion

The results consistently show that intra-device similarity is higher than cross-device similarity across participants, indicating that the dynamic characteristics of a signature are influenced by the acquisition device and its interaction conditions. This behavior is consistent with the prior literature reporting that feature consistency and verification performance can degrade under changes in acquisition conditions, including device-related variability [6,8,22,23].

In particular, the Galaxy Ultra presented lower intra-device DTW distances than the Tab S6 Lite. While this work does not explicitly measure hardware latency or sampling rate, such differences can plausibly affect temporal–spatial stability in pen-based input. Similar sensitivity of online signature features to acquisition characteristics and device conditions has been discussed in studies focusing on feature consistency and robustness [6,22,23]. These group-level observations are consistent with the verification trends observed in the ROC-based analysis, where cross-device conditions exhibit a degradation in discriminability, reinforcing the impact of device mismatch on online signature verification performance [8,22,23].

Cross-device evaluations showed a clear performance degradation, supporting the interpretation that device changes introduce systematic distortions in signature trajectories and kinematic patterns. Under the threat model considered in this pilot study, an adversary does not need to compromise the sensor; instead, risk emerges when enrollment and verification occur on different devices and genuine-score variability increases under mismatch. In this setting, broader genuine-score dispersion can reduce separability from impostor scores and make operating-threshold selection less stable across platforms. These effects may arise from differences in writing area, interaction mechanics, and sensing characteristics across devices. Device-induced distortions and their impact on performance have been reported in mobile signature verification and broader state-of-the-art analyses [8,22,23].

Despite the small sample size, participant-level analyses showed consistent patterns: each user maintained a characteristic signing style, although the degree of stability varied across devices. This observation aligns with classical results in the signature verification literature emphasizing individuality and the persistence of writer-specific traits, even under variability sources [7,8,26]. At the same time, the present study has clear scope limitations: it is exploratory, includes a limited number of participants and sessions, and is restricted to two devices within the same stylus ecosystem. Therefore, the findings should be interpreted as evidence of device-dependent effects in this controlled pilot context, not as universal performance bounds across all mobile platforms. Overall, the experiment confirms that device dependency remains a major source of variability in online signature verification, motivating cross-device normalization or adaptation strategies for reliable authentication in realistic mobile environments, consistent with prior robustness discussions under device mismatch [8,22,23].

## 6. Conclusions

In this work, cross-device variability in online signature dynamics was analyzed using consumer mobile devices equipped with stylus input. Signatures captured and compared on the same device exhibited lower DTW distances, while higher variability was observed under cross-device conditions, indicating a measurable influence of device form factor and writing surface characteristics on recorded trajectories. These results were consistent at both the descriptive distance level and the verification level, where cross-device scenarios reduced separability between genuine and impostor comparisons in ROC-based analysis [8,22,23]. From an operational perspective, this behavior is relevant to mobile biometric authentication workflows in which enrollment and verification may occur on different devices, since device mismatch can reduce threshold stability and decision reliability across platforms. The present contribution is exploratory and limited to a pilot setting (limited participants, sessions, and two devices in a common stylus ecosystem); therefore, conclusions should be interpreted as evidence of device-dependent effects in this context rather than as universal performance limits. Even with this scope, the proposed acquisition and analysis pipeline provides a practical basis for future studies with larger cohorts and broader device heterogeneity. Overall, the findings support the need to explicitly account for cross-device variability when designing and evaluating online signature verification systems intended for real-world mobile deployment.

The presented analysis highlights the importance of considering device-dependent effects when designing online signature verification systems based on consumer mobile platforms. In practical deployments, thresholds and models tuned for a single device may not transfer reliably to heterogeneous devices, potentially increasing false rejections or false acceptances when enrollment and verification occur on different platforms [8,22,23]. The proposed acquisition and analysis framework provides a practical basis for future studies involving larger datasets, more diverse user populations, and extended acquisition protocols that include additional sources of variability, such as different stylus types, screen sizes, or sensing behaviors.

Finally, beyond performance degradation, the consistent increase in cross-device DTW distances suggests that signatures acquired across devices should not be treated as interchangeable samples during enrollment or statistical modeling without explicit compensation for device mismatch. Overall, these findings emphasize the need to account for device-related variability when designing and evaluating online signature verification systems intended for cross-device deployment. Future work may explore cross-device normalization through geometric coordinate normalization to a device-independent writing frame, temporal resampling/alignment to reduce sampling-rate and speed differences, and lightweight score-level calibration or domain-adaptation mappings to improve interoperability across platforms [8,22,23].

## Figures and Tables

**Table 1:** Comparison of related work in online signature analysis.

Reference	Input Device	Features	Method	Cross-Device	Dataset
[20]	Pen-based digitizer	XY time series	DTW	No	Not specified
[4]	Pen-based device	XY, dynamic features	HMM	No	Public (MCYT [3], SVC2004 [1])
[22]	Mobile signing devices	Robust feature sets	Feature-based evaluation	Yes	Not specified
This work	Consumer mobile	XY, pressure	Multivariate DTW distance analysis	Yes	Private

## Data Availability

Data are available on request from the corresponding author. The data are not publicly available due to privacy considerations.
